# Discrepancy Between Liquid Biopsy and Tumor Next-Generation Sequencing Due to Low Tumor Fraction in a Patient With Lung Adenocarcinoma

**DOI:** 10.7759/cureus.70119

**Published:** 2024-09-24

**Authors:** Madeline C Baker, Trevor A Rose, Bruna Pellini

**Affiliations:** 1 Morsani College of Medicine, University of South Florida, Tampa, USA; 2 Radiology, H. Lee Moffitt Cancer Center and Research Institute, Tampa, USA; 3 Thoracic Oncology, H. Lee Moffitt Cancer Center and Research Institute, Tampa, USA; 4 Department of Oncologic Sciences, University of South Florida, Tampa, USA

**Keywords:** circulating tumor dna (ctdna), liquid biopsy, metastatic adenocarcinoma of lung, next generation sequencing (ngs), non-small cell lung carcinoma (nsclc), tumor fraction

## Abstract

Tumor next-generation sequencing (NGS) is the gold standard molecular testing for driver genomic alterations in patients with advanced non-small cell lung cancer (NSCLC). However, it requires a biopsy, which is an invasive procedure. In contrast, a liquid biopsy is a minimally invasive test that measures circulating tumor DNA (ctDNA) in the plasma and can be also used for molecular profiling. We report a case of a patient with stage IV metastatic lung adenocarcinoma with a negative liquid biopsy for tumor-derived genomic alterations but positive tissue NGS for mutations, including a driver *KRAS^G12C^* mutation. The discrepancy between the two results can be attributed to low levels of ctDNA determined by tumor fraction below 1%, which prevents the liquid biopsy assay from detecting genomic alterations when the tumor shedding into the blood is below the detection threshold. It is well known in the literature that false negative liquid biopsies are possible, but a significant finding this case highlights is the clinical importance of tumor fraction in a liquid biopsy report. We conclude that patients with a liquid biopsy with low tumor fraction need further testing with tumor NGS to determine the presence of driver genomic alterations.

## Introduction

There are two major ways of testing for oncogenic driver mutations in patients with non-small cell lung cancer (NSCLC). The first approach is a tumor tissue-based assay that requires a tumor biopsy and uses next-generation sequencing (NGS) to identify genomic alterations. The second is a liquid biopsy, a less invasive test that measures circulating tumor DNA (ctDNA) in blood plasma [1]. A liquid biopsy has a quicker turnaround time for delivering results, but it can give false negative results. If a liquid biopsy is used, the ctDNA levels can serve as a guiding factor to determine whether tumor NGS is needed to confirm the results [2]. Recently, tumor fraction has emerged as a means of quantifying ctDNA levels and has been shown as a prognostic biomarker across many types of advanced cancers [3]. Utilizing tumor fraction values has the potential to guide clinical decision-making and treatments. We report a case of a patient with stage IV metastatic lung adenocarcinoma with false negative liquid biopsy results due to low levels of ctDNA determined by tumor fraction less than 1%.

## Case presentation

**Figure 1:**
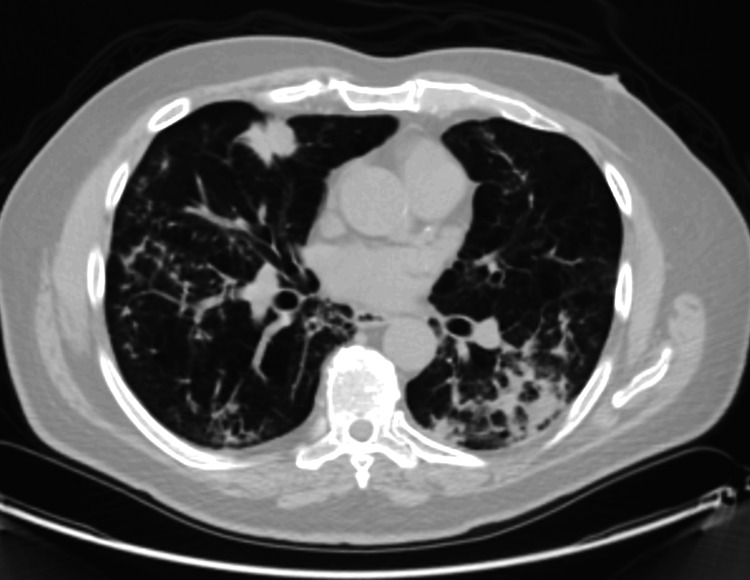
Chest CT November 2023: Before Treatment Abbreviations: CT, computed tomography

A 71-year-old male with a history of heavy tobacco smoking was diagnosed with stage IV right lower lobe adenocarcinoma of the lung. In late November of 2023, he underwent a Positron Emission Tomography-Computed Tomography (PET-CT) and brain Magnetic Resonance Imaging (MRI) for staging. Imaging revealed metastasis in the left lung, mediastinal lymph nodes, and bones. He then underwent a CT-guided lung biopsy that revealed the diagnosis of lung adenocarcinoma (Figure 1). A liquid biopsy, tumor tissue NGS, and programmed death ligand 1 (PD-L1) testing were also ordered. When presenting to the thoracic clinic to start treatment in early December of 2023, his clinical status had deteriorated, and he was requiring oxygen through a nasal cannula. Chest imaging was obtained during an urgent care visit, and it was suggestive of bilateral lymphangitic spread. Tumor NGS results were still pending, and his liquid biopsy (FoundationOne® Liquid CDx; Foundation Medicine, Inc., Cambridge, MA, USA) showed a low ctDNA tumor fraction (<1%) and detectable mutations in *DNMT3A* and *TET2* suspicious for clonal hematopoiesis. Due to his clinical deterioration, systemic therapy had to be started while awaiting tumor tissue NGS results. He then received one cycle of intravenous pemetrexed at a regimen dose of 400 mg/m^2^ and carboplatin at an area under the concentration-time curve of 5 mg/mL/min with significant improvement of his respiratory symptoms. The pemetrexed was dose reduced by 20% due to a creatinine level of 1.7 mg/dL.

**Figure 2:**
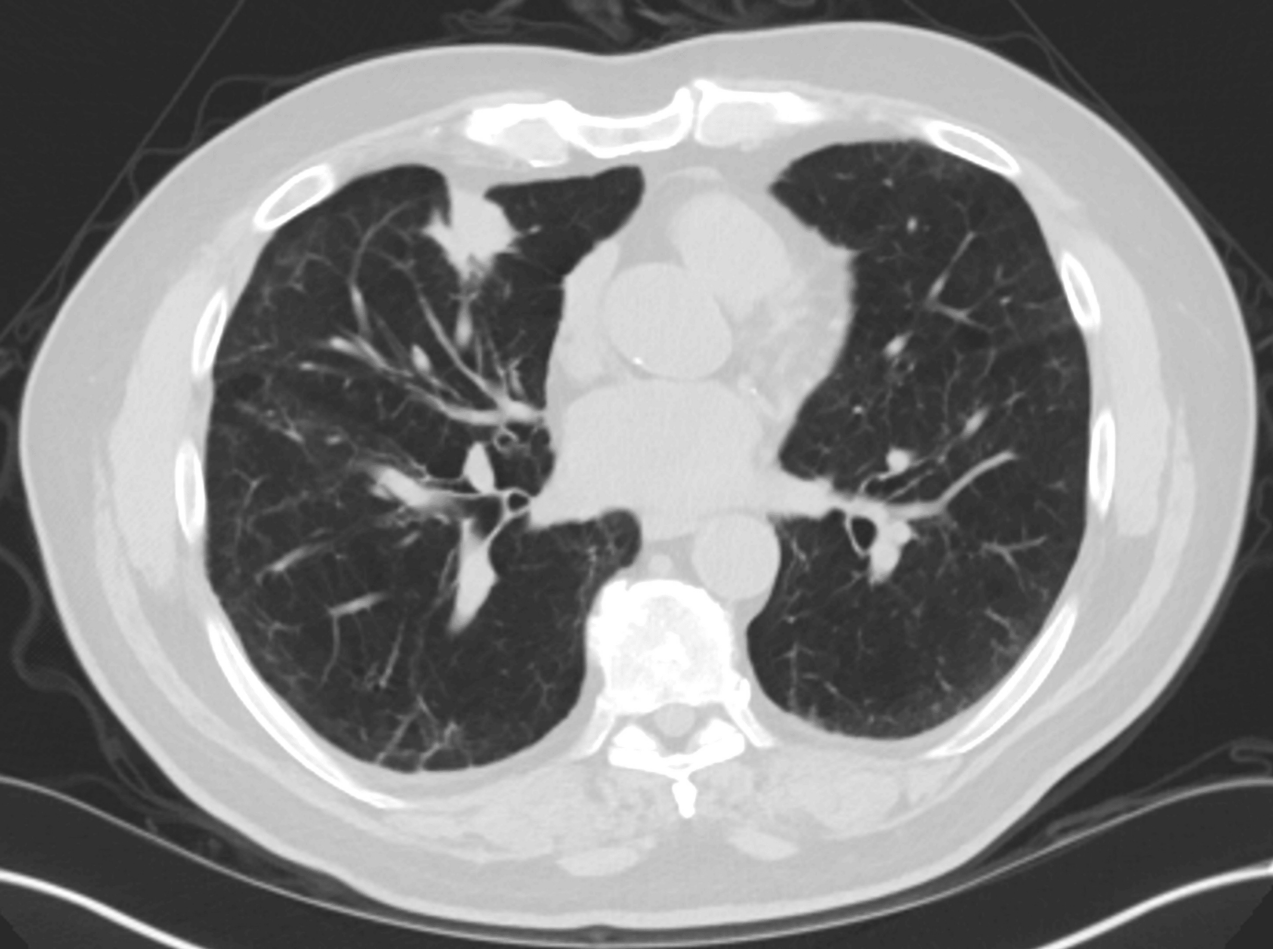
Chest CT January 2024: After Two Cycles of Treatment Abbreviations: CT, computed tomography

In mid-December 2023, before cycle two of systemic therapy, the tumor NGS testing had resulted. His Moffitt STAR 2.0 Cancer Mutation and Molecular Biomarker Profile (Tampa, FL, USA) showed a *KRAS^G12C^*mutation and co-mutations in *KEAP1*, *CDKN2A*, and *STK11* (Table 1). A high tumor mutation burden was also noted with 14.1 mutations/megabase (Table 1). The plan was to add pembrolizumab to his treatment in cycle two. However, his creatinine level was elevated at 2.8 mg/dL, so pemetrexed was held, and he was treated with carboplatin and pembrolizumab 200 mg. Body imaging was obtained after two cycles of treatment, showing significant improvement of his bilateral lymphangitic spread, stable right upper lobe nodule, and no evidence of new metastatic lesions (Figure 2). He then continued treatment achieving stable disease in subsequent imaging.

**Table 1:** FoundationOne®Liquid CDx Results vs. Moffitt STAR 2.0 Cancer Mutation and Molecular Biomarker Profile Abbreviations: NGS, next-generation sequencing; ctDNA, circulating tumor DNA; TMB, tumor mutation burden; Muts/Mb, mutations per megabase; PD-L1 TPS, programmed death ligand 1 tumor production score; N/A, not applicable

	Mutations	ctDNA Tumor Fraction	TMB (Muts/Mb)	PD-L1 TPS
Liquid Biopsy	DNMT3A E599fs*49, TET2 E330*	Low (<1%)	3	N/A
Tumor NGS	KRAS G12C, KEAP1 E343*, CDKN2A D84Y STK11 Y36_Q37delinsC*, TP53 Q165*	N/A	14.1	N/A
PD-L1 (223C)	N/A	N/A	N/A	0%

## Discussion

This case report highlights the importance of ctDNA tumor fraction to inform clinicians on the high probability of a false negative liquid biopsy result. Several studies have reported on the liquid biopsy false negative rates in patients with advanced non-small cell lung cancer [4]. However, until the advent of tumor fraction, clinicians did not have a way to determine if a liquid biopsy result was truly negative vs. falsely negative. In our clinic, we have several patients in which tumor fraction assisted us in determining the need to wait for a tumor tissue NGS or not. One of our other patients, a 71-year-old woman with metastatic adenocarcinoma of the right lung, had a liquid biopsy with ctDNA tumor fraction of <1% with a detectable mutation in *TP53*. Her tumor NGS showed a *KRAS*^*G12D*^mutation and co-mutations in *CTNNB1* and *ARID1A*. This is a very similar scenario to the patient discussed in this case report, again highlighting the importance of tumor fraction reporting. In contrast, a high ctDNA tumor fraction may indicate that the liquid biopsy is more reliable for detecting the presence or absence of driver mutations. For example, an 81-year-old female presented to the thoracic clinic with metastatic squamous cell carcinoma of the right lung. The liquid biopsy showed an absence of driver mutations and a high tumor fraction of 8.2%. In this case, the liquid biopsy matched the tumor NGS, which confirmed the absence of known driver mutations. One limitation of this report is its focus on a single case and a small number of patient examples, which may limit the generalizability of the findings. Larger, prospective studies with more diverse patient populations can be used to further validate these observations.

Our patient cases suggest that ctDNA tumor fraction is helpful in determining the likelihood of a false negative vs. a truly negative liquid biopsy result. Clinicians must be cautious when relying on a negative liquid biopsy with a low tumor fraction to determine the absence of driver mutations. It is recommended to perform tumor and blood molecular profiling when possible [5]. However, in situations where resources are limited, we suggest prioritizing tumor tissue NGS for patients with liquid biopsies with low tumor fraction. 

## Conclusions

This case report suggests that clinicians can assess the reliability of a liquid biopsy to determine the presence of driver genomic alterations based on tumor fraction levels in NSCLC patients. If the tumor fraction is below 1% and the liquid biopsy is negative, tumor NGS is warranted given a high probability of a false negative result. We urge liquid biopsy companies to make tumor fraction reporting standard, considering how impactful this information can be for clinical decision-making. In the context of NSCLC, accurate assessment of tumor fraction is particularly crucial due to the potential for significant implications on treatment choices and patient outcomes.
